# RNAScope-Ancestry: A Cross-Modality Framework for Inferring Genetic Ancestry from RNA-Seq with Application to MECA

**DOI:** 10.21203/rs.3.rs-7801062/v1

**Published:** 2026-03-08

**Authors:** Rashi Verma, Shivam Sharma, Harriet NA Blankson, Emine Guven, Andrea Pearson, Charles D. Searles, Peter Baltrus, Tene T. Lewis, Priscilla Pemu, Dean Jones, Arshed Ali Quyyumi, Herman Taylor, I. King Jordan, Robert Meller

**Affiliations:** Morehouse School of Medicine; Georgia Institute of Technology; Morehouse School of Medicine; Morehouse School of Medicine; Morehouse School of Medicine; Atlanta VA Health Care System; Morehouse School of Medicine; Emory University School of Medicine; Morehouse School of Medicine; Emory University School of Medicine; Emory University School of Medicine; Morehouse School of Medicine; Georgia Institute of Technology; Morehouse School of Medicine

**Keywords:** Genetic Ancestry, Cardiovascular Disease, Short-Read RNA-Seq, Race, Ethnicity

## Abstract

Genetic ancestry inference traditionally relies on genotyping arrays or whole-genome sequencing. We present RNAScope-Ancestry, a computational pipeline that leverages short-read RNA-seq for dual-purpose ancestry and transcriptomic analysis. Using RNA-seq from 490 MECA participants and 1000 Genomes reference populations, we performed variant calling, quality filtering, principal component analysis and retained 230 high-frequency SNPs for ancestry estimation via Rye algorithm for ancestry inferences. MECA participants aligned with African and admixed populations, predominantly West African. Correlation with gene expression identified the top 50 ancestry-associated transcripts. Validation in 109 longitudinal samples confirmed reproducibility. The pipeline is open-source and generalizable: https://github.com/rob-meller/; https://github.com/Vermarashi/.

## Introduction

Genetic ancestry analysis has traditionally relied on genotyping arrays and whole-genome sequencing that enable high-resolution inferences of genetic variation by capturing a comprehensive set of variants across the genome [1, 2]. These approaches are well-validated and widely used for ancestry estimation due to their robustness and accuracy. In contrast, RNA sequencing (RNA-seq), primarily designed for transcriptomic profiling, offers an alternative approach by simultaneously quantifying gene expression and identifying genetic variants. Although RNA-seq does not surpass traditional methods in variant resolution, it presents distinct advantages, particularly in leveraging existing transcriptomic data to infer genetic ancestry.

RNA-seq is widely used and readily available in many genomic studies, making it a valuable resource for genetic ancestry analysis without requiring additional sequencing efforts. Moreover, the integration of genetic ancestry inferences with transcriptome data providing a complementary dimension that captures gene expression patterns influenced by both genetic background and environmental factors. RNA-seq data, with its ability to capture the full spectrum of transcript variants, presents a unique opportunity to enhance the resolution and accuracy of genetic ancestry analyses [3]. This dual functionality makes RNA-seq particularly valuable in studies of complex diseases, where gene expression data can provide deeper insights into population-specific genetic traits and their functional implications.

The application of RNA-seq for genetic ancestry analysis remains relatively unexplored [4-6]. Thus, the study presents RNAScope-ancestry to assesses the genetic ancestry of MECA study participants, providing a high-resolution view using short-read RNA-seq data. By contextualizing genetic and clinical findings within African, European, and American ancestries, it offers insights into cardiovascular health disparities among Black adults. By integrating genetic ancestry into CVD research, we can better understand how gene-environmental interactions shape health outcomes. This understanding can guide targeted interventions to reduce CVD disparities and promote health equity.

## Results

### Characteristics of MECA dataset

The study includes a diverse cohort of Black adults (n=490, visit 1; n=109, visit 2). It encompasses a broad age range (>36) and both genders, with detailed demographic, clinical, and behavioral data, including blood pressure, cholesterol, BMI, glucose, HbA1c, C-reactive protein, lifestyle factors (diet, exercise, smoking, alcohol, sleep), psychosocial measures (stress, resilience), and environmental data (geographic location, healthcare access). Longitudinal follow-up tracks cardiovascular risk trajectories [8].

### Selection of high-quality SNPs

High-quality RNA-seq data is essential for accurate SNP inference. From the Visit 1 dataset, 18.86M variants were identified and filtered to 2.59M SNPs, while the reference dataset was reduced from 82.49M to 81.57M variants. Comparative analysis revealed 1.26M common SNPs, further refined through quality control and LD pruning to 1,415 (Visit 1) and 163,279 (reference) independent variants. The intersection yielded 230 common variants for PCA and ancestry analysis, ensuring robust results (Figure 2).

### Inference of ancestry from RNA-seq data

The genetic relationship among MECA participants and reference samples from four regional ancestry groups, computed using PCA, are shown in Figure 3A. The PCA plot illustrates distinct clustering patterns for individuals from various ancestral backgrounds based on principal components (PC1 and PC2). Individuals of European ancestry are clearly separated along PC1, whereas African ancestry forms a tight cluster closer to the origin, with admixed and Native American populations distributed between these clusters. The MECA participants predominantly overlap with the African ancestry cluster, suggesting that most of these individuals are of African descent, with a few showing potential admixture.

Ancestry proportions were quantified using Rye algorithm. African reference populations principally display African ancestry, with negligible contributions from European or American ancestries (Figure 3B). Conversely, European reference populations are primarily European. Admixed individuals exhibit varying proportions of African, European, and Native American ancestries, reflecting the complexity of their genetic backgrounds. MECA study participants exhibit ancestry proportions like admixed individuals, with significant African and European contributions. These analyses highlight the utility of PCA and admixture estimation in elucidating the ancestry patterns of diverse populations and contextualizing participant genetic profiles. The ancestry estimates are consistent with participants’ self-identified ethnic backgrounds, which is a second level of ethnic identity beneath the ethnic group designation

### Inference of sub-ancestry from RNA-seq data

Next, we were able to assess the genetic structure of sub-African populations and their relationship to the study participants. This is critical not only to test the sensitivity of our approach but also for interpreting genetic associations within the MECA cohort. Consequently, it establishes the participant’s primary African genetic ancestry with highlighting any admixture as well. Understanding this genetic structure enhances the context of our analyses and aligns the study's aim to investigate ancestry driven cardiovascular health in a population using short read RNA-Seq data analysis.

PCA plots across the top four PCs showed enhanced population clustering patterns with HWE cutoffs from 1x10^−4^ to 0.4. At a cutoff of 1x10^−4^, reference populations formed poorly differentiated clusters and showed highly distant separation from the participant population along PC1 (Figure 4A; B). Interestingly, the plots for PC2, PC3, and PC4 revealed clear clustering of reference populations, with the participant population closely aligning with Western African populations, particularly ESN and YRI (Figure 4**C; D)**. As we applied HWE cutoff 0.4, clustering patterns along PC1 remain the same. However, PC2, PC3, and PC4 clearly depicted the participant population's distribution across Western African populations, suggesting the filtration of probable artifacts from the data (Figure 4**E; F**). These findings emphasize the sensitivity of PCA clustering to HWE thresholds, illustrating how parameter selection can impact population differentiation and the identification of genetic structure.

Ancestry proportion showed largely East African ancestry in the East African group and largely West African ancestry in the West African group. The MECA participants exhibited a mixed ancestry pattern, with a predominant contribution from West African populations and minimal contribution from East African populations (Figure 5).

### Genes Correlated to Ancestry

Correlation analysis between CPM values and ancestry fractions of European, African, and American populations did not reveal any significant associations. However, further analysis focusing specifically on Sub-Saharan African ancestry uncovered distinct gene sets associated with East and West African ancestry fractions. Among the 13,566 significant genes, 54 genes exhibited positive correlations with West African ancestry, while 10,488 genes showed positive correlations with East African ancestry. We reported top 50 genes with the highest positive and negative correlations with East and West African ancestry (Figure 6). Moreover, the genes with the strongest correlations (both positive and negative) demonstrated highly significant −log10(adjusted p-values), further emphasizing their robust association with the respective ancestry fractions.

### Performance accuracy with dataset 2

We analyzed the data from Visit 2 participants that were aligned closely with their corresponding groups in Visit 1, validating the method's accuracy in assigning ancestry (Figure 7A). African ancestry dominates both visits, with 106 and 108 participants, respectively, while European and American ancestries show negligible representation. (Figure 7B). The decrease in the number of European participants from Visit 1 to Visit 2 could be attributed to technical variations or sample reprocessing. The near-identical distributions reinforce the reproducibility of the methodology when applied to subsets of the same population. PCA plots for Sub-Saharan African ancestry results aligns well with the findings from the Visit 1 dataset (Figure 7C; D). Ancestry fraction for visit 2 revealed the African ancestry (Figure 7E) and West-African ancestry (Figure 7F) as primary component with minimal contributions from other ancestries. This uniform pattern across visits underscores the method's reliability in detecting and quantifying ancestry proportions consistently.

## DISCUSSION

The application of bioinformatics tools and computational approaches in understanding genetic ancestry represents a significant advancement in the field of genomics. While genotyping arrays and whole-genome sequencing remain the gold standard for ancestry inference, RNA-Seq technologies have emerged as a complementary approach. These advancements are driven by their ability to generate high-throughput data, genome-wide coverage, enhanced accuracy, ability to analyze admixed population, flexibility in analysis and adaptability for studying ancient DNA [6, 18-20]. This leads to enhance detection of complex genetic variations, including structural variants, haplotype phasing, and isoform diversity, which are crucial for accurate ancestry analysis. Although RNA-Seq does not provide the same variant resolution as traditional methods, its widespread availability and routine use in transcriptomic studies make it a valuable resource for ancestry analysis.

By leveraging genetic variants embedded within transcriptomic data, RNAScope-Ancestry allows for ancestry inference without the need for additional genomic sequencing, making it a cost-effective alternative in studies where DNA-based data may not be available. Furthermore, its application extends to admixed populations, where it can aid in identifying population-specific expression patterns influenced by genetic ancestry. This approach also facilitates the investigation of how ancestry-related genetic factors contribute to gene regulation, enhancing our understanding of complex traits and disease susceptibilities across diverse populations.

We aimed to explore ancestry-driven cardiovascular insights in participants of MECA study using short-read RNA-Seq data. To achieve this, we developed a systematic workflow to assess and stratify the genetic makeup of MECA participants against reference populations from Admixed, Europe, America, and Africa. Additionally, we focused on their Sub-Saharan African genetic background by comparing the cohort with various African reference populations. Our approach was further validated using data from participants who attended a second visit, as well as a subset from the initial visit. Principal Component Analysis (PCA) revealed distinct clustering patterns, revealing predominance of African ancestry within our MECA participant cohort.

Study revealed diverse genetic makeup of MECA participants with clustering near African, and Admixed groups. At the sub-African level, we observed that MECA participants predominantly clustered with West African (Niger-Congo) ancestry, along with a minor East African component. The East African component primarily reflects Bantu ancestry, which originated from the southwestern regions of Africa during the Bantu expansion, not directly from East Africa. The LWK sample from Kenya, which was used to represent East African ancestry, is more accurately described as Bantu due to its linguistic and genetic connection to the Bantu-speaking populations of Central and Southern Africa. This distinction is important because most enslaved Africans brought to the Americas came from West and Central Africa, not East Africa. Therefore, while it is correct to label the LWK sample as East African geographically, referring to it as Bantu ancestry provides a more accurate historical and genetic context. This clarification aligns with the historical migration patterns and enhances the precision of our findings, reflecting the complex heritage of African Americans.

Correlation analysis depicted top 50 genes significantly correlated to sub–Saharan African ancestries. Noticeably, the genes exhibit a positive correlation with East African ancestry and an equally strong negative correlation with West African Ancestry, with an adjusted p-value indicating high statistical significance (**Suppl. Fig. 1**). The observed correlations follow a predictable pattern due to the inherent non-independence of ancestry components. Since the sum of ancestry proportions is constrained to 100%, a positive correlation for one ancestry component necessarily implies a negative correlation for the other. This non-independence acts as an internal control, ensuring that our results are consistent and correctly interpreted. Such patterns have been noted in previous ancestry studies and are a fundamental property of proportional data. To address this dependency, we recalculated East and West African ancestry proportions as unscaled absolute values by multiplying total African ancestry with their respective relative proportions, thereby removing artificial dependencies and preserving the natural structure of the data (Figure 6).

We validated our RNAScope-Ancestry pipeline using data from MECA Participants who returned for a second visit [n (visit 2 = 109]. The validation confirms the reliability of our approach that reproduces consistent ancestry distributions across related cohorts and self-identified ethnic backgrounds. Although all participants self-identified as African American, our genetic ancestry analysis revealed substantial sub-continental African heterogeneity, including contribution from west and east African and European ancestries. These findings support the observation that self-identified race doesn’t align with genetic ancestry. Therefore, biomedical research relying solely on race labels overlook biologically relevant ancestry-related variations. Integrating ancestry analysis provides a more accurate framework for interpreting gene expression pattern in diverse population [21].

### CHALLENGES:

The genetic ancestry analysis of MECA samples using short-read RNA-seq data presented several challenges, particularly in achieving proper clustering in the PCA plots along PC1. Initial PCA plots exhibited significant clustering for reference populations; however, the MECA samples displayed an unusual spread, indicating potential noise or confounding factors in the dataset (**Suppl. Fig. 2A; B**). To address this, we implemented a series of filtering steps, beginning with LD pruning. Despite this, PC1 and PC2 remained suboptimal for the MECA samples (**Suppl. Fig. 2C**) while PC1 and PC3 demonstrated better clustering for some residual noise (**Suppl. Fig. 2D**). Then, we filter variants with missing genotype >10% across all samples and Hardy-Weinberg threshold (1x10^−4^). These adjustments effectively removed low-quality variants and potential artifacts, thereby enhancing the resolution of genetic structure (Figure 3A). The improved separation in PCA plots following LD pruning and HWE filtering highlights the importance of minimizing noise and ensuring data quality.

Next challenge we faced with sub-Saharan African ancestry analysis likely due to the presence of most common variants present across these populations. Initial PCA results revealed dispersed clustering of MECA samples (**Suppl. Fig. 3**) which suggested the necessity of incorporating rare variants to improve clustering accuracy. To address this, imputation was incorporated into the pipeline which improved clustering on PCA plots, particularly for PC2, PC3, and PC4 (Figure 4**C-D**). Further refinement with HWE thresholds resulted in better alignment of participant samples with reference populations (Figure 4**E; F**). Notably, the MECA query samples appear to shift towards the European pole along PC1 (Figure 4A-B). This shift makes sense, as these samples might have a genetic composition that is aligned with European populations. As a separate query group, the MECA samples are not admixed per se but may share genetic features associated with European ancestry, which is reflected in their position along the principal component axis. The shift along PC1 further supports the validity of our results and underscores the importance of considering the genetic background of the query samples in interpreting the PCA plots.

While genome-wide studies generally require hundreds of thousands to millions of variants, however, our analysis demonstrated that even a small subset of variants (n<500) can effectively capture significant ancestry-informative signals, particularly in admixture populations. Winkler et al. highlighted that enough ancestry-informative markers can effectively support genome-wide scans for disease associations, particularly in admixed populations [22]. Notably, the alignment of PCA clustering with self-identified ethnicities underscores the utility of this approach, suggesting that a well-curated set of variants can provide meaningful insights into genetic ancestry. This finding highlights the potential of short-read RNA sequencing data, which, despite its lower variant count, can yield valuable genetic information. Our results advocate for the use of smaller variant panels in genetic ancestry studies, especially when cost-effective methods like short-read RNA sequencing are preferred over whole-genome sequencing, offering an accessible and efficient alternative for research on genetic admixture.

These results also have profound implications for genetic epidemiology and precision medicine. Given that MECA study participants exhibit genetic affinities with specific West African population, it is crucial to acknowledge these subpopulations in future genetic studies and healthcare applications. Furthermore, the genetic data presented here highlights the importance of utilizing ancestry-informative markers in Mende and Yoruba West groups that could be valuable for refining genetic models to predict disease risk and enabling more targeted and personalized healthcare strategies. Additionally, this study also highlights the need to diversify genomic databases to better represent African genetic diversity, as African populations have been historically underrepresented. Expanding the inclusion of groups will improve the generalizability of research findings and help create more accurate genetic models, benefiting African descent populations worldwide.

While this study utilizes RNA-seq data to infer genetic ancestry, it is important to acknowledge that RNA-seq is primarily designed for gene expression analysis rather than ancestry estimation. Unlike genotyping arrays or whole-genome sequencing, RNA-seq-based ancestry inference may be influenced by several factors, including expression variability, batch effects, and sample-specific biases. Additionally, gene expression is context-dependent and may vary across tissues, environmental conditions, and disease states, which could introduce variability in ancestry estimations. Limited reference populations may not fully represent East African diversity, highlighting the need for broader datasets in genetic databases. Usage of small number of variants may reduce the resolution of ancestry inference compared to studies utilizing high-density genotype data. Replicating these findings by addressing limitations in independent datasets will strengthen the evidence for ancestry, ancestry-related expression differences and its health implications.

Despite the limitation, our findings exhibit strong consistency with established ancestry patterns, reinforcing the robustness of the approach. The workflow demonstrates the efficacy of filtering and utilizing high-quality SNPs to infer genetic ancestry in a diverse cohort of MECA participants. It highlights genetic variation that influences gene expression offering insights into the biological processes linked to genetic ancestry. It generates consistent results from one over another dataset that make it more reliable when analyzing diverse populations. It allows the integration of ancestry results with gene expression analysis which provides complementary layer of information that makes short RNA-seq particularly useful in studying traits or diseases influenced by regulatory mechanisms. By integration with genetics data, the workflow can provide a more comprehensive understanding of how ancestral origins shape gene expression, offering a deeper perspective on the complex interplay between genetics and phenotype.

## Conclusion

RNAScope-Ancestry demonstrates that short-read RNA-seq can be repurposed beyond transcriptomics to provide reliable genetic ancestry inference, linking population structure with gene expression. By validating the pipeline in MECA participants and ensuring reproducibility across visits, our framework establishes a scalable, generalizable approach for ancestry-aware analyses, particularly in underrepresented populations. This method enables integrative, dual-purpose studies that can advance precision medicine, population genetics, and complex disease research using existing RNA-seq datasets.

## METHODOLGY

**Figure 1:**
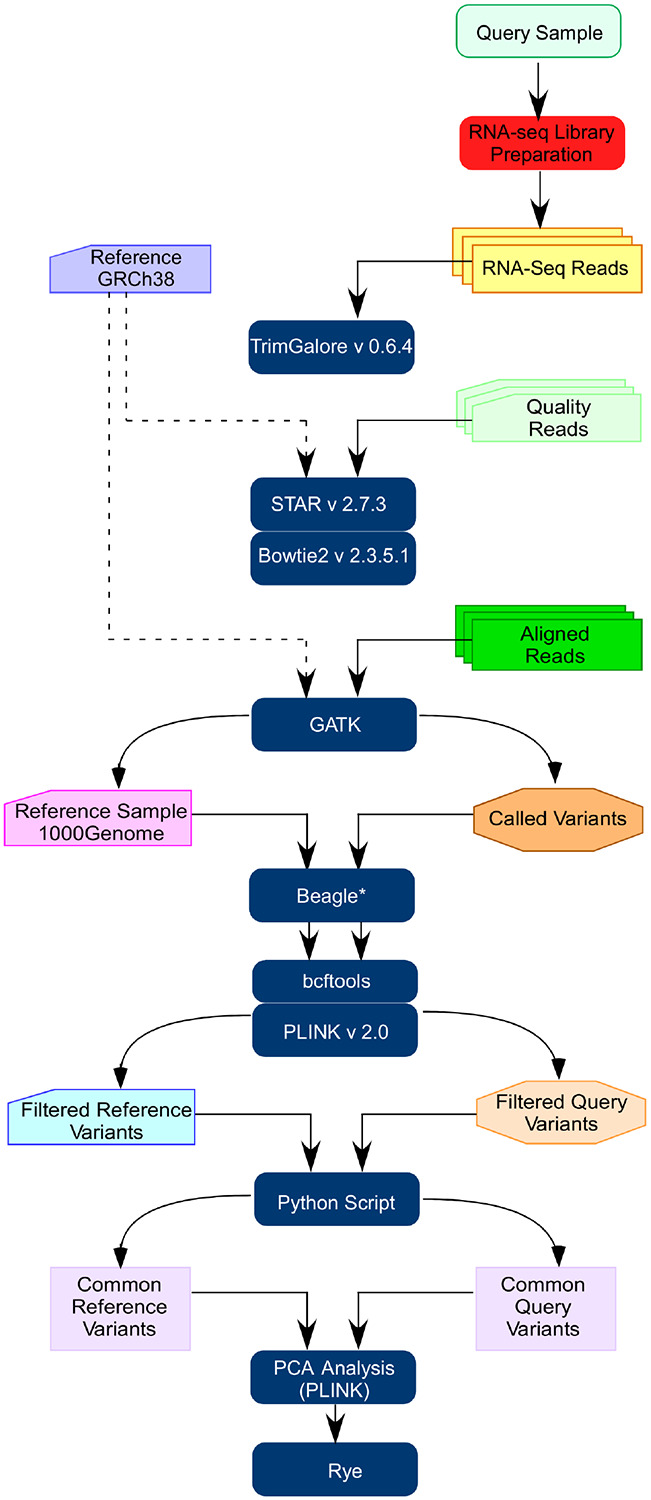
Overview of the genetic ancestry analysis pipeline using RNA-seq data. The schematic illustrates the step-by-step protocol employed to infer genetic ancestry from RNA sequencing, including variant calling, filtering, and downstream population structure analysis.

**Figure 2:**
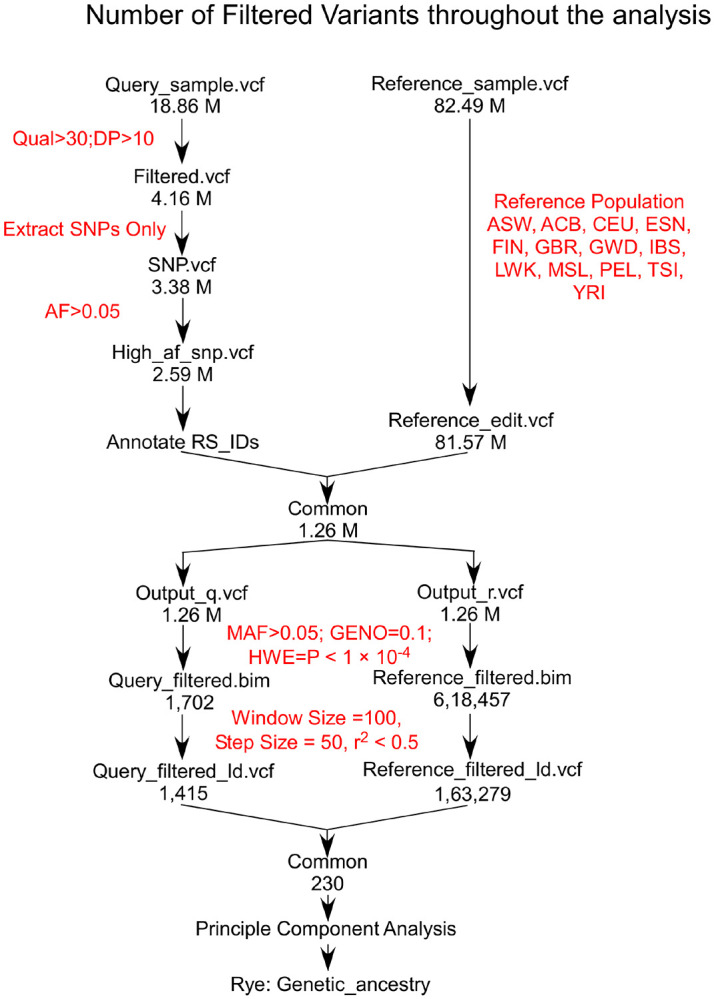
Variant filtering progression across the analysis pipeline. The figure presents the number of variants retained after each major filtering step, highlighting the impact of quality control, Hardy-Weinberg equilibrium testing, and linkage disequilibrium pruning on the dataset.

**Figure 3:**
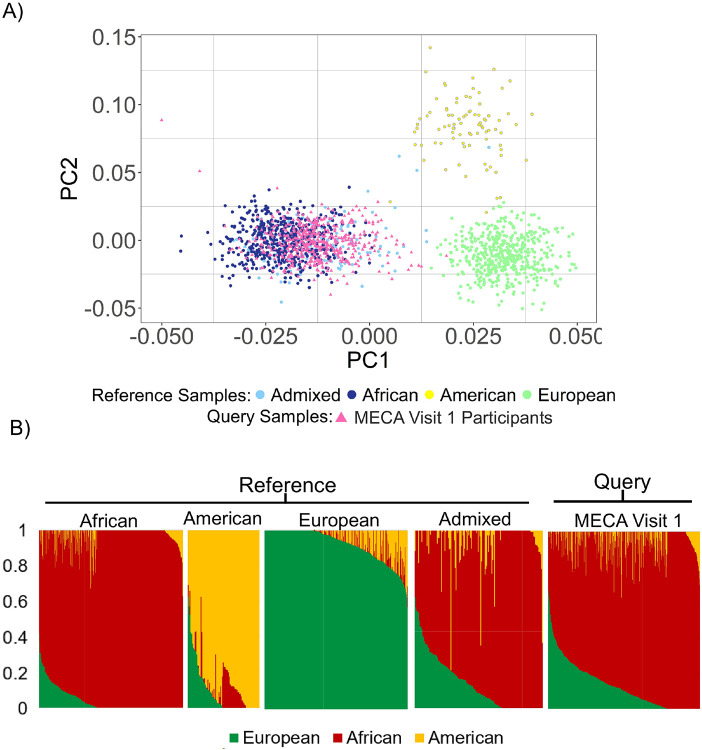
Genetic ancestry inferences on MECA participants (visit 1). **(A)** PCA of MECA participants (hot pink) and ancestry group reference samples (colored as shown). (**B)** Ancestry and admixture patterns for MECA participants. Ancestry fractions (colored as shown) are indicated for each participant. All groups are scaled based on the number of participants.

**Figure 4:**
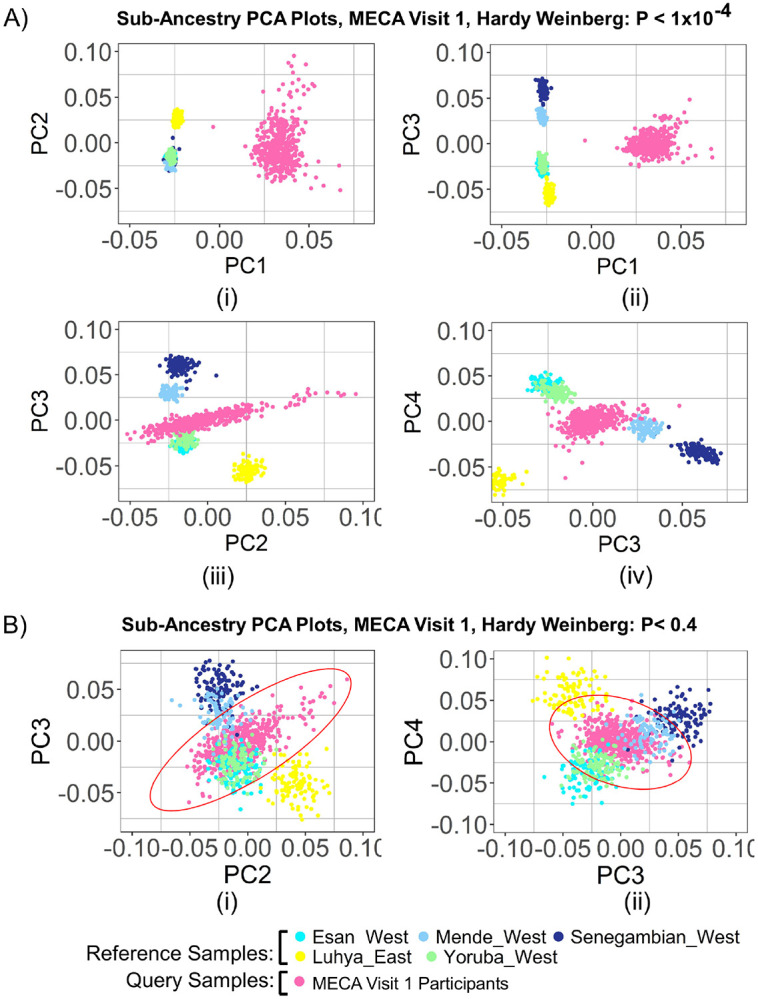
PCA analysis illustrating the effect of Hardy-Weinberg Equilibrium filtering thresholds on Sub-Saharan African ancestry clustering. **(A)** PCA plots generated after applying a HWE filter (P < 1×10^−4^), showing clustering patterns based on (i) PC1 vs PC2, (ii) PC1 vs PC3, (iii) PC2 vs PC3, and (iv) PC3 vs PC4. Study participants (visit 1) and reference populations clustered separately. **(B)**PCA plots generated after applying a HWE threshold (P < 0.4), showing (i) PC2 vs PC3 and (ii) PC3 vs PC4. PC1 vs PC2 and PC1 vs PC3 are not shown due to negligible differences from plots in section **A**. The ancestry clusters, particularly for MECA participants (visit 1), appear closer and show partial overlap with reference populations, providing enhanced resolution of subtle ancestry patterns.

**Figure 5:**
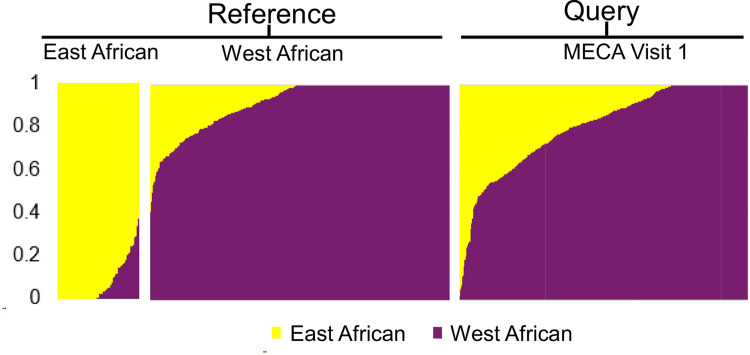
Sub-Sharan African Ancestry and admixture patterns for MECA participants. Ancestry pattern of MECA participants (visit 1) is predominantly aligns with west African reference samples. Ancestry fractions (colored as shown) are indicated for each participant. All groups are scaled based on the number of participants.

**Figure 6:**
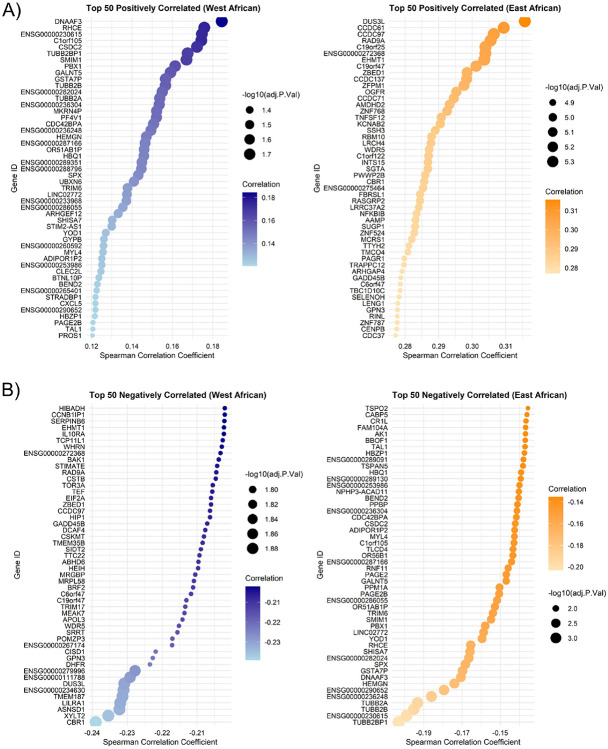
Gene correlation with Sub-Sharan African Ancestry. **(A)** Top 50 positively correlated genes to East and West African Ancestry (visit 1). **(B)** Top 50 negatively correlated genes to East and West African Ancestry (visit 1).

**Figure 7:**
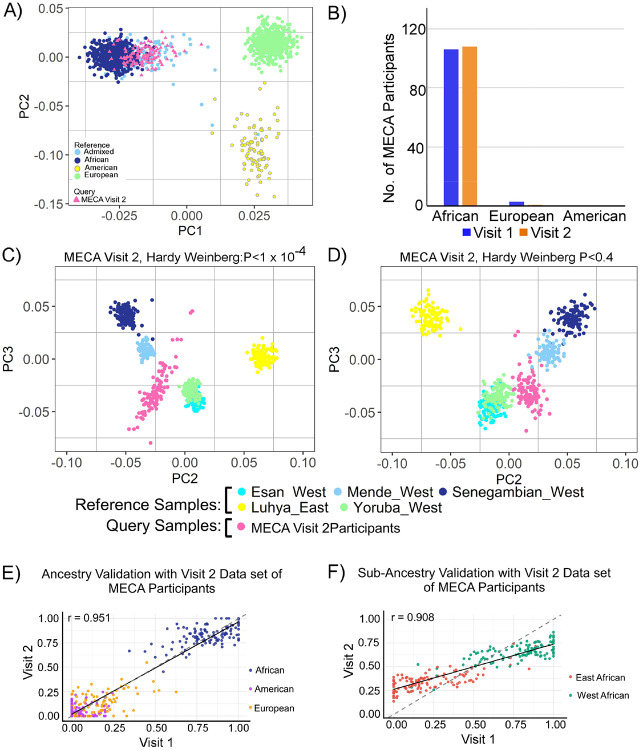
Validation of genetic ancestry analysis pipeline with MECA participants (visit 2). **(A)** PCA of MECA participants visit 2 (hot pink) and ancestry group reference samples (colored as shown). **(B)** Number of participants present in visit 1 and visit 2. **(C)** PCA plots highlighting Sub-Sharan African Ancestry using Hardy-Weinberg Equilibrium (HWE) filtering thresholds (P < 1×10^−4^). **(D)** PCA plot using a HWE threshold (P < 0.4), showing comparatively tighter clustering and partial overlap. **(E)**Correlation analysis of overall ancestry proportions between MECA participants in Visit 1 and Visit 2. (F) Correlation of sub-Saharan African ancestry estimates between Visit 1 and Visit 2, supporting consistency and robustness of the ancestry inference pipeline.

The RNAScope-Ancestry pipeline starts with sample collection and RNA-Seq library preparation. Figure 1 highlights the steps of protocol. We applied this framework to the available participants of Morehouse-Emory Cardiovascular Center for Health Equity (MECA) study [7]. The Visit 1 dataset was used for ancestry estimation, while the Visit 2 dataset (comprising subsequent visits of participants already included in the Visit 1 dataset) validated the findings.

### Data and Sample Collection

A multi-faceted approach was employed to investigate cardiovascular health disparities among Black adults in Atlanta, classifying neighborhoods as "at-risk" or "resilient" based on cardiovascular outcomes hospitalization [8]. Over 1,400 individuals were surveyed, with 599 participants undergoing clinical evaluations and blood collection across two visits (Visit 1: n=490; Visit 2: n=109).All eligible participants in this cohort self-identified as Black or African American adults, aged 18+, residing in targeted neighborhoods, with exclusions for non-residency, inability to consent, or conditions interfering with assessments [8].Additionally, for the intervention subset, individuals who could not adhere to the eHealth tools or coaching protocols due to technological or other barriers were excluded. These criteria ensured a representative sample while maintaining the study's scientific rigor and relevance. This protocol ensured a robust cohort for examining genetic and environmental factors influencing cardiovascular risk and resilience.

### RNA-Seq Library Preparation

Blood was collected into PAXgene Blood RNA tubes (Pre Analytix, Qiagen) and the RNA was extracted using the MagMAX for Stabilized Blood Tubes RNA Isolation Kit, compatible with PAXgene Blood RNA Tubes (ThermoFisher Scientific). RNA quality was assessed using a Fragment Analyzer (Agilent) and then one microgram of total RNA was subjected to globin transcript depletion using the GLOBINclear Kit, human (ThermoFisher Scientific). Ten nanograms of the globin-depleted RNA were used as input for cDNA synthesis using the Clontech SMART-Seq v4 Ultra Low Input RNA kit (Takara Bio) according to the manufacturer’s instructions. Amplified cDNA was fragmented and appended with dual-indexed bar codes using the Nextera XT DNA Library Preparation kit (Illumina). Libraries were validated by capillary electrophoresis on a TapeStation 4200 (Agilent), pooled at equimolar concentrations, and sequenced with PE100 reads on an Illumina NovaSeq 6000, yielding ~30 million reads per sample on average.

### Data Alignment and Variant Calling

The sequenced data were trimmed (TrimGalore v0.6.4) and aligned to the GRCh38 human reference genome (STAR v2.7.3a and Bowtie2 v2.3.5.1) [9, 10]. Aligned BAM files were sorted and indexed (SAMtools v1.10) [11]. Variant calling was performed (GATK pipeline) [12]. PCR duplicates were marked (MarkDuplicates), spliced alignments processed (SplitNCigarReads), and base quality scores were recalibrated (BaseRecalibrator) to correct systematic sequencing errors. HaplotypeCaller was used in GVCF mode for variant calling, and the resulting GVCF files were jointly genotyped in GenomicsDB (GenotypeGVCFs). Variants were filtered and recalibrated variant quality score (ApplyVQSR).

### Preprocessing of MECA and References Samples

Variants were annotated (bcftools) with databases such as dbSNP138, HapMap, and Mills and 1000G indels. Quality filters were applied, retaining only non-singleton variants with quality scores > 30 and depth > 10. Normalization was conducted to ensure consistent representation of variants. Genotype data from the MECA samples were designated as “query samples.”

Reference data (African, American, European, Admixed = 1249) from the 1000 Genomes Project served as “reference samples” (**Table 1**). Native American ancestry is represented by Peruvian (PEL) samples, which have been shown to carry a high proportion of Native American ancestry (>80%) as reported in Conley et al., 2023 [13]. African ancestry is represented by Esan (ESN), Gambian (GWD), Luhya (LWK), Mende (MSL), and Yoruba (YRI) samples. European ancestry is represented by Utah residents (CEU), Finnish (FIN), British (GBR), Iberian (IBS), and Toscani (TSI) populations. Admixed American, including African Caribbean in Barbados (ACB) and African Americans in the Southwest U.S. (ASW), are categorized as such due to their known two-way African European admixture. While these populations are sometimes classified under African ancestry in other studies, we use the admixed category to better reflect their genetic background. This classification allows for a more accurate interpretation of genetic structure in our study cohort.

Chromosome names were standardized, and variants were normalized to a biallelic format. Quality control steps included filtering for minor allele frequency (MAF < 0.05), genotyping rate (0.1), and Hardy-Weinberg equilibrium (P < 1 × 10^−4^). To reduce redundant variants, linkage disequilibrium (LD) pruning was performed (100:50:0.5). The resulting intersected variants from query and reference datasets were merged and converted into PLINK binary format (**Script**).

### Ancestry Estimation

PCA was conducted using PLINK (v2.0) to detect genetic structure and estimate ancestry proportions among MECA participants [14]. Ancestry was estimated with Rapid ancestrY Estimation (Rye), an efficient algorithm that leverages principal components for robust ancestry inference [13]. The final dataset included reference groups from the 1000 Genomes Project categorized as African, European, and American. A population-to-group mapping file aggregated populations into these continental groups: African (ESN, GWD, LWK, MSL, YRI), European (CEU, FIN, GBR, IBS, TSI), American (PEL), and Admixed (ACB, ASW) (**Table 1**). Rye analysis used 30 principal components allowing robust ancestry fraction estimation for each participant in the MECA study.

### Sub-African Ancestry Analysis

Phasing and genotype imputation were performed using Beagle v5.1 for the Sub-Saharan African ancestry analysis of the MECA sample [15]. Phasing involved the reconstruction of chromosomal phases for variants, which improved the accuracy of imputation. Imputation used the phased haplotypes to infer missing genotypes by referencing haplotypes from the 1000 Genomes Project, resulting in a complete and high-quality dataset for downstream ancestry analysis. Sub-Saharan African populations—West African (ESN, YRI, GWD, MSL) and East African (LWK)—were utilized as references (**Table 1**). The quality control process included filtering, linkage disequilibrium (LD) pruning, merging of common variants, and performing principal component analysis (PCA). Ancestry proportions were calculated using Rye.

### Gene Expression Correlation Analysis

Next, we aimed to identify genes significantly correlated with ancestry fractions in the dataset. Libraries were quantified using StringTie2 [16]. Gene expression was calculated as fragments per kilobase of transcript per million mapped reads (FPKM) that were normalized using the trimmed mean method. The counts per million (CPM) were computed for each gene across all samples. The CPM matrix was then utilized to calculate Spearman correlation coefficients between CPM values and ancestry fractions. Since ancestry proportions are constrained to sum to 100%, the correlations for East African ancestry would be the exact inverse of East African ancestry. To address potential dependencies between ancestry components, we calculated East and West African ancestry as unscaled absolute values based on the total African ancestry and their relative proportions. To account for multiple testing, adjusted p-values were determined using the Benjamini-Hochberg method [17]. For visualization, we selected the top 50 genes with the highest positive and strongest negative correlations.

### Method Validation

We validated our approach for ancestry analysis by applying it to the Visit 2 dataset participants. The same parameters were used to check the reproducibility of the proposed approach. Approach accuracy was measured by comparing the ancestry PCA plot and fractions calculated for participants of visit 1 to visit 2.

## Supplementary Material

This is a list of supplementary files associated with this preprint. Click to download.

- TABLE1.docx
- SUPPLEMENTARYDATAlegends.docx
- SupplFig.pdf

Table 1 is available in the Supplementary Files section.
